# The Impact of Cognitive Reserve on Cognitive Functions in the Elderly: A Neuropsychological Investigation

**DOI:** 10.1002/alz.092995

**Published:** 2025-01-03

**Authors:** Daphne Gasparre, Elisabetta Ricciardi, Chiara Abbatantuono, Laura Prontu, Federica Marocchino, Madia Marika Biasi, Giulia Paparella, Alessandro Introna, Alessandro Caffò, Maria Fara De Caro, Paolo Taurisano

**Affiliations:** ^1^ University of Bari Aldo Moro, Bari Italy; ^2^ University of Cagliari, Cagliari Italy

## Abstract

**Background:**

Cognitive reserve (CR) represents the susceptibility to age‐related brain changes or Alzheimer’s disease‐related pathology. It is the ability to withstand the effects of aging and neurological damage. Several factors influence CR, including education, work, activites, mental activity, and social networks, which play a crucial role in an individual’s ability to preserve the global cognitive functioning and counteract cognitive decline. Boosting cognitive reserve has become a relevant focus in our efforts. In this study, we aim to investigate how cognitive reserve could influence the cognitive decline in the elderly.

**Method:**

Targeted neuropsychological assessment were administered to patients with Mild cognitive Impairment and dementia. We analyse through software R 4.3.2., the relationship between of cognitive reserve and other cognitive functions such as attention and memory capabilities.

**Results:**

Our preliminary findings suggest a clear correlation between higher cognitive reserve and improved performance on these tests. This connection appeared particularly significant in the early stages of deterioration (Mild Cognitive Impairment). In other words the notion of cognitive reserve posits the prospect of interventions capable of mitigating cognitive aging or diminishing the susceptibility to dementia.

**Conclusions:**

CR has a key role across the aging. In particular individuals with MCI could prevent and contrast cognitive decline boosting their CR, for example doing more activities, learning new language, gaining new skills, doing physical activities. Further research is needed to fully grasp this relationship and devise methods to extend cognitive reserve in order to promote cognitive health.

